# Basic Concepts on the Role of Nuclear Factor Erythroid-Derived 2-Like 2 (Nrf2) in Age-Related Diseases

**DOI:** 10.3390/ijms20133208

**Published:** 2019-06-29

**Authors:** Fabiane Valentini Francisqueti-Ferron, Artur Junio Togneri Ferron, Jéssica Leite Garcia, Carol Cristina Vágula de Almeida Silva, Mariane Róvero Costa, Cristina Schmitt Gregolin, Fernando Moreto, Ana Lúcia A. Ferreira, Igor Otávio Minatel, Camila Renata Correa

**Affiliations:** 1 Medical School, São Paulo State University, Botucatu 18618-970, SP, Brazil; 2 Institute of Biosciences, São Paulo State University, Botucatu 18618-689, SP, Brazil

**Keywords:** Nrf2, age-related disorders, oxidative stress

## Abstract

The transcription factor Nrf2 (nuclear factor erythroid 2-related factor 2) is one of the most important oxidative stress regulator in the human body. Once Nrf2 regulates the expression of a large number of cytoprotective genes, it plays a crucial role in the prevention of several diseases, including age-related disorders. However, the involvement of Nrf2 on these conditions is complex and needs to be clarified. Here, a brief compilation of the Nrf2 enrollment in the pathophysiology of the most common age-related diseases and bring insights for future research on the Nrf2 pathway is described. This review shows a controversial response of this transcriptional factor on the presented diseases. This reinforces the necessity of more studies to investigate modulation strategies for Nrf2, making it a possible therapeutic target in the treatment of age-related disorders.

## 1. Introduction

Aging is a highly complex process influenced by genetic and environmental factors, which can be defined as the progressive loss of an organism’s optimal function until its eventual failure and death. Many aging-associated disorders, as soon as the senescence process, are involved with perturbed energy balance. For example, the peripheral signals (insulin, ghrelin, cholecystokinin, and adipokines (leptin, adiponectin, resistin)) together with the central regulatory inputs (primarily via the hypothalamus) are impaired in aging. Moreover, neurodegenerative disorders characterized by cognitive and/or motor symptoms that progressively worsen over time, are also common in elderly people, leading to reduced quality of life, increased medical costs and eventual death [1].

Among the several pathways associated with age-related disorder development, both oxidative/electrophilic and inflammatory stresses play important roles [2,3]. Within this perspective, studies aiming to mitigate or attenuate these processes have been exhaustively performed [4]. In the last years, novel strategies have been raised as promising, such as the regulation of gene expression [5] and stimulation of key proteins related to oxidative and inflammatory responses control [6]. One of these proteins is the nuclear factor erythroid-derived 2-like 2 (Nrf2) [7], a nuclear factor constitutively expressed in the cytosol and inhibited by its negative regulator Kelch-like ECH (Enoyl-CoA Hydratase)-associated protein 1 (Keap1) [8].

**Figure 1:**
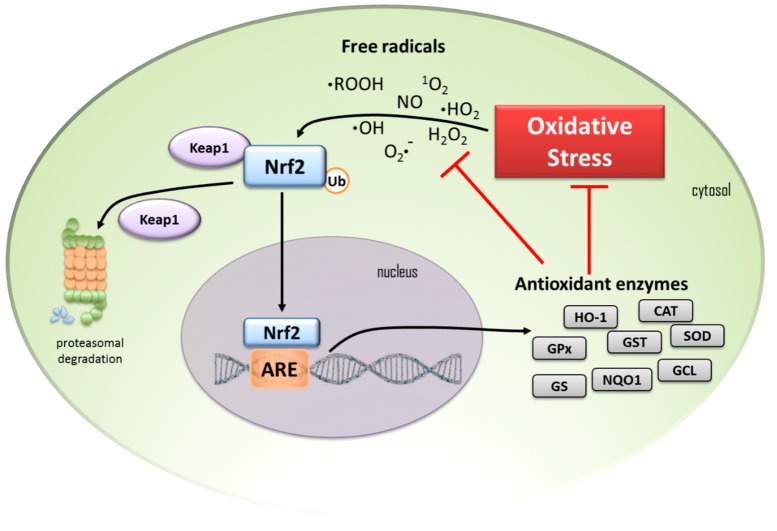
In cells under homeostatic conditions, cytosolic transcription factor Nrf2 is kept at low levels by proteasomal degradation trigged by the Keap1 protein complex. When cells are under oxidative stress, free radicals induce the Nrf2 to release from Keap1, escaping from proteasomal degradation, and it translocates to the nucleus. In the nucleus, Nfr2 binds to the ARE and starts the transcription of antioxidant enzymes as heme oxygenase-1 (HO-1), glutathione peroxidase (GPx), glutathione S-transferase (GST), superoxide dismutase (SOD), catalase (CAT), glutathione reductase (GR), NAD(P)H:quinone oxidoreductase 1 (NQO1), glutamine-cysteine ligase (GCL), and glutathione synthetase (GS). These enzymes act by reducing the cell oxidative stress and free radicals. Black arrows indicate pathways activation; Red T-bars indicate blocking processes.

The most studied biological process under Nrf2 control is the redox homeostasis. During the redox homeostasis, the persistence of the Keap1-Nrf2 association in the cytosol leads to the ubiquitination of this complex and consequent degradation by the proteasome. Conditions of redox imbalance induce the dissociation of the Keap1-Nrf2 complex, releasing the nuclear factor that translocate to the nucleus, while the inhibitory component Keap1 is degraded via ubiquitin-proteasome [7,9]. Inside the cell nucleus, Nrf2 is coupled to the gene region associated with the antioxidant-response element (ARE) expression. This mechanism is responsible by the expression of many antioxidant and detoxification genes. It includes the production of enzymes such as hemeoxigenase 1 (HO-1), catalase (CAT), superoxide dismutase (SOD), and glutathione peroxidase (GPx), the expression of phase II detoxifying enzymes, such as glutathione S-transferase (GST), and enzymes responsible for the glutathione tripeptide (GSH) synthesis, such as glutamine-cysteine ligase (GCL) and glutathione synthetase (GS) [9] (Figure 1). The AREs were primarily described as xenobiotic-responsive elements [10], which present an upper expression induced by planar aromatic compounds. At the same time, one more accurate name was coined, electrophile-response element (EpRE) [11], and is actually an appropriate synonym for AREs [12,13].

However, in the last decade, several mechanisms related to the Nrf2 activation and controlled by this transcriptional factor have been described. At the same time, the literature is controversial regarding its participation on age-related disorders. Here we describe a brief compilation of the Nrf2 contribution in the pathophysiology of the most common age-related diseases and bring insights for future research about the pathways which involve Nrf2.

## 2. Hypertension

Hypertension is characterized by increased systolic and/or diastolic blood pressure [14] and considered one of the most common chronic non-transmissible diseases able to lead to vasculature and central nervous system changes. This condition has a multifactorial etiology which includes smoking, diet, genetics, family history, and preexisting pathologies [15]. However, oxidative and nitrosative stress appear to be a common feature in hypertensive disorders responsible by an impairment of physiological functions, as well as cell signaling, promoting vascular damage, a common condition in the hypertensive state [16].

The main cause of hypertension is the inappropriate activation of the renin-angiotensin system (RAS), once angiotensin II and associated RAS are involved in the regulation of blood pressure, vasoconstriction, sodium intake, and potassium excretion [17]. At the same time, angiotensin II increases the expression of NADPH oxidase and the generation of ROS, potential mediators of some renin-angiotensin-induced hypertension effects [17]. Angiotensin II can activate the NADPH system, which increases the generation of ROS, inactivating the NO and generating peroxynitrite. This condition leads to an impairment of the NO-dependent endothelial vasodilatation and to an uncoupling of endothelial nitric oxide synthase, which generates additional superoxide production and contribute to the oxidative stress increase [18,19]. In this scenario, there is a large Nrf2 migration to the nucleus in response to the pro-oxidant environment. However, the nuclear accumulation of Nrf2 hyper-regulates the expression of angiotensin that potentiates the angiotensin II signaling, enhancing the oxidative stress [20,21]. In this context, in a study involving a hypertensive rat model [22] has suggested that hypertension could be one of the causes of Nrf2 misregulation and not the opposite. The findings suggest that the Nrf2 antioxidant defense system would not be sufficient to attenuate the oxidative stress effects, possibly due to the elevated levels of Nrf2 repressors in hypertensive animals.

Considering that the excessive production of reactive oxygen species (ROS) and deregulation of the antioxidant defense system can lead to endothelium cellular damage and dysfunction, studies are necessary to focus on alternative mechanisms intrinsic to upstream and downstream molecules associated with a defective Nrf2 signaling system. Thus, enhancing Nrf2 activity may have a therapeutic potential for ameliorating hypertension.

## 3. Type-2 Diabetes

Diabetes mellitus (DM) is a chronic metabolic disorder from genetic and/or environmental etiology characterized by increased levels of blood glucose due the impairment in insulin production or its secretion/action [23]. DM is a major health problem that comprises more than 400 million people diagnosed worldwide and the projection of more than 500 million by 2035. This rise is mainly due aging, unhealthy diets, physical inactivity, overweight and obesity [24]. During aging, there is an impairment in the glucose tolerance which makes evident some diabetic characteristics, such as post-prandial glycemia [25]. It is estimated that more than half of Americans over 65 years old have pre-diabetes (impaired glucose tolerance) and one-third have type-2 diabetes (T2DM) [26]. Oxidative stress is an important mediator in the pathogenesis, complications, and progression of DM.

In type-2 diabetes (T2DM), insulin resistance promotes β-cell failure through mitochondrial stress, which increases the reactive species production, leading to cellular damage [27]. In addition, β-cells are particularly susceptible to oxidative damage due to the presently low levels of expression and antioxidant activity [28,29]. Although cytosolic superoxide dismutase is normally expressed in the pancreatic islets, catalase and glutathione peroxidase levels are reduced compared to other tissues [30]. This scenario is the major trigger for the progressive loss of β-cell mass observed in T2DM.

The Nrf2/keap1/ARE pathway is the main redox homeostasis mediator [31]. This redox signaling pathway regulates several genes associated with oxidative stress and studies have shown that Nrf2/keap1/ARE is the main β-cell protective mechanism [32]. The Nrf2 depletion decreases the expression of antioxidant genes, exacerbating the oxidative damage; in the opposite, the genetic or pharmacological activation of Nrf2 in *db*/*db* mice suppress T2DM development and reactive species accumulation, DNA adducts formation and β-cell apoptosis [33,34].

In addition to the positive effect on β-cells, the Nrf2 pathway demonstrates the influence of insulin resistance. Nrf2 activation improved the insulin resistance and decreased the blood glucose levels in Keap1 knockout mice [33]. Other studies show that Nfr2 inducers improve systemic insulin resistance in experimental diabetes and obesity models and increase glucose uptake [35,36]. A recent study also suggests that the decreased oxidative stress in the hypothalamus due the increased Nrf2 signaling can improve insulin resistance [37].

Moreover, in a condition of constant hyperglycemia advanced glycation end-product (AGE) formation occurs, resulting from the non-enzymatic reaction between a sugar and an amino group of proteins. AGEs are involved in many biological reactions, such as endocytic uptake and degradation, oxidative stress, and cytokine induction due the interaction with cellular receptors, including the receptor of advanced glycation end-products (RAGE) [38]. AGE/RAGE binding is one pathway involved in the oxidative stress increase; the stimulation of pro-inflammatory and pro-coagulant agents is also cited as a main pathogenic cause of vascular disorders in diabetic individuals [39]. The enzyme glycoxalase I (Glo I) is a detoxifier of methylgyoxal (MGO), which consequently inhibits the formation of AGE. The overexpression of this enzyme by Nfr2 reduces the hyperglycemia-induced AGE as previously demonstrated [40,41]. Furthermore, hyperglycemia induces mitochondrial reactive species overproduction and the antioxidant agents upregulated by the Nrf2/Keap1/ARE pathway was demonstrated to be a more efficient detoxifier than the classic low molecular-weight antioxidants [42,43].

Since oxidative stress is a pivotal factor in the DM pathogenesis and complications, studies have emerged aiming to increase the antioxidant response and reducing the oxidative impacts by the investigation Nrf2 activators [34,44,45,46]. In vitro studies, animal models, and clinical trials suggest that the up-regulation of the Nrf2 pathway can be protective against DM (T1 and T2) by suppressing the disease progression and preventing complications [32,47,48,49]. This evidence encourages the activation of Nrf2 as a target against diabetes; however, more studies are necessary to evaluate this effect.

## 4. Cataract

The eye is a prominent oxidative stress target organ since it is continually exposed to many oxidative conditions, such as photo-oxidation, ionizing radiation, smoke, and several forms of pollutants. The retina is one of the most vulnerable ocular regions due to high metabolic activity, becoming a highly perfused and oxygenated tissue. The retina also contains higher concentrations of polyunsaturated fatty acids than other tissues in the human body [50]. All these factors make it vulnerable to oxidative actions, such as reactive oxygen species (ROS). Thus, oxidative stress has been associated with many ocular disorders, among them, the cataract [51].

Cataract is a form of blurred vision that results from the cloudiness of the lens, being the most common cause of vision loss in people over 40 years old around the world [52]. There are three main types of cataracts: subcapsular, cortical, and nuclear cataracts, each one with different associated risk factors [51]. Nonetheless, aging and oxidative stress, such as that which occurs due to ultraviolet irradiation, are the common denominators [53].

The human lens consists of a, b, and c crystalline proteins, and oxidative stress may lead to the protein aggregation, developing clumps, which results in loss of transparency and cataract. Together with oxidation of crystalline proteins, the DNA damage, the membrane lipid peroxidation, and the unbalance in calcium homeostasis are all contributors for cataract formation. This illness is also prevalent in other diseases that have oxidative stress in the physiopathology, such as diabetes [53,54].

The imbalance between reactive species and antioxidant protection, defined as oxidative stress, is an important condition associated with age-related cataract formation. Within this context, the Keap1-Nrf2-ARE system is the center of the antioxidant response regulation, responsible for the control of several cytoprotective proteins at the transcriptional level [55]. ROS overproduction leads to the suppression of Nrf2-dependent antioxidant protection in lens epithelial cells [56]. A drastic decrease in the Nrf2 level (protein and gene) significantly increases the Keap1 level (protein and gene), and highly elevated levels of DNA demethylation in the Keap1 promoter were found in human lens epithelial cells culture, human aging lenses, and diabetic cataractous lenses [57].

In opposition, in clear human lenses and cultured lenses, DNA methylation was demonstrated as a promoter of Keap1 gene demethylation, a crucial mechanism for cataract formation in an age-dependent behavior [58]. This process of demethylation accelerates Nrf2 proteasomal degradation [57] and impairs Nrf2 antioxidant activity, leading to cataract formation [58]. These findings show that Nrf2 inducers may also act as anti-cataract formation compounds [59].

## 5. Bone Metabolism Disorders

Bone formation is a complex process that occurs throughout an individual’s life. Bones constantly change due to two main processes: modeling and remodeling. The modeling process is responsible by the formation of new bone in response to environmental forces, resulting in a new bone shape. Bone remodeling consists in the old bone tissue removal to be replaced for a new one, an essential process for bone homeostasis. Therefore, reduced bone remodeling or an imbalance between bone resorption and formation is associated with some age-related bone disorders, such as osteoporosis [60]. Epidemiological reports from the World Health Organization shows that in 50 year-old women, the fracture rate is about 40 and the risk increases with age [61].

Multiple pathogenetic mechanisms are responsible for bone mass loss and skeletal microarchitectural deterioration, such as excessive bone resorption or inadequate bone formation in response to the increased resorption during bone remodeling. Moreover, studies have demonstrated an important contribution from the redox imbalance to bone, once the reduced antioxidant levels would enhance bone resorption whereas the reduction in oxidative stress may provide protection against osteoporosis in the aged [61].

The role of Nrf2 in osteoblast differentiation and activity is still controversial and dependent of some factors, such as age, sex, genetic, and physiological or pathological conditions. Studies suggest that Nrf2 is required not only for normal postnatal bone acquisition [62], but some data also show that Nrf2-deficient osteoblasts lose their ability for differentiation and mineralization [63]. In osteoblast progenitor cells, the role of Nrf2 may be associated with the intracellular level of ROS, which are elevated in Nrf2-deficient stromal cells [63]. Increased ROS in oxidative stress condition inhibit the osteoblast differentiation [64]. In opposition, osteogenesis depends of low physiological amounts of reactive species. The bone morphogenetic protein 2 (BMP2) is responsible by promoting osteoblast progenitor cells to mature osteoblasts in a mechanism NOX4 (NADPH oxidase 4) dependent via ROS production [65]. Also, the osteoprogenitor cells differentiation produces hydrogen peroxide, which is fundamental for the adequate mineralization and osteogenic marker genes expression [66]. These data suggest that the relationship between osteoblastogenesis and Nrf2 or ROS is rather complex. In MC3T3-E1 osteoblastic cells, the Nrf2 overexpression causes deleterious effects because it inhibits Runx2 [67]. Runx2 is a master transcription factor that regulates both embryonic bone development and postnatal osteoblastic function. Based on this evidence, some researchers believe that Nrf2 might inhibit osteoblastogenesis [68].

However, the fundamental participation of Nrf2 in bone formation has been demonstrated in situations of fracture repair in response to mechanical loading. Under the condition of fracture repair Nrf2 activation occurs. On the other hand, in Nrf2 knockout mice, both bone healing and recovery of mechanical strength are impaired, probably due the reduction in vascular endothelial growth factor (VEGF) [69]. Thus, these data suggest an essential role of Nrf2 in bone regeneration.

Nrf2 signaling also influences the regulation of osteoclast formation and activity. The receptor activator of nuclear factor kappa-Β ligand (RANKL) is the main inducer of osteoclast differentiation. In condition of overexpression of Nrf2 occurs an enhancement of RANK ligand which suppress osteoclast differentiation; however, the deletion of Nrf2 reduces the antioxidant enzymes and elevates the intracellular ROS, leading to an increase in osteoclast number and stimulation of osteoclast activity [70]. Together, these findings show an indirect effect of Nrf2 on osteoclasts formation and activity. Moreover, Nrf2 is also able to interfere with the actin ring, affecting the osteoclast activity. This actin ring is a sealing zone present in mature osteoclasts and crucial for bone resorption. Yet, Nrf2 deficiency leads to the actin ring formation induced by the RANK ligand and bone resorption, suggesting that the bone resorption normal range is dependent of Nrf2 [68].

Therefore, Nrf2 exerts a critical role in the regulation of bone homeostasis. However, it is important to emphasize the participation of endocrine organs on bone tissues and cells health. However, Nrf2 may be a pharmacological target for bone integrity maintenance in pathological situations.

## 6. Alzheimer’s Disease

Alzheimer’s disease (AD) is a common age-related neurodegenerative disorder characterized by the progressive learning and memory impairment. The main hallmarks of AD are: senile plaques, which are extracellular accumulations of amyloid beta (Aβ) peptide; and neurofibrillary tangles, which are composed of hyper phosphorylated tau protein [71]. According to the Aβ cascade hypothesis, this substance is the main cause of neurotoxic injuries in AD, activating many biochemical pathogenic mediators, among them oxidative stress and synaptic dysfunction, leading to AD [72,73]. However, the exact mechanisms associated with dementia remain unclear.

Increased ROS in oxidative stress conditions are important mediators of AD. At the same time that the brain consumes high amounts of oxygen, it has a limited antioxidant defense, becoming a sensitive organ to oxidative stress [74]. The Nrf2 is a key redox-regulated gene with a critical role against oxidative stress, and the level of Nrf2 in the nucleus is decreased in neurological disorders, such as AD [75]. Recently, the literature reports that Nrf2 is able to regulate different endogenous redox-regulated enzymes, as the heme oxygenase-1 (HO-1) and glutathione cysteine ligase modulatory subunit (GCLM) via phosphorylated phosphatidylinositol 3-kinase, phosphorylated Akt, and phosphorylated glycogen synthase kinase 3 beta (p-PI3K/Akt/GSK3β) pathway. This mechanism has an important role in many signaling functions, being investigated also in AD brain and AD mouse models [76,77,78,79,80].

Notably, HO-1 activation has some benefits, including learning and memory improvement [75,81] and studies report that Nrf2 is able to increase the HO-1 expression [82]. In both in vitro and in vivo studies, the high expression of Nrf2 was associated with decreased Aβ-induced neurodegeneration and oxidative stress [83]. Corroborating this result, Kanninen et al. showed that the overexpression of Nrf2 improves spatial learning and memory in a mice model of AD [80,84].

Although the results show a protective effect of Nrf2 against AD, Ramsey et al. reported that the level of Nrf2 is different according to brain location: usually this transcription factor is found in both nucleus and cytoplasm; however, in AD patients is primarily present in the cytoplasm [85]. Moreover, both activation and gene expression are decreased in AD, consistent with Nrf2 level changes [83]. However, some researchers show an opposite result, with an up-regulation in AD brains compared to control [86,87,88,89]. These controversial results are also affected by the disease stage and the studied brain region [90]. Within this context, the Nrf2 activation seems to exert a protective role against AD-related pathophysiology, being a possible target for drug development against AD.

## 7. Parkinson’s Disease

Parkinson’s disease (PD) is a progressive, incurable, and age-related disease affecting 1.8% individuals by the age of 65 years [91] that presents, as major clinical hallmarks, resting tremor, rigidity, postural instability, and akinesia, accompanied by cognitive impairment [92]. The PD etiology comprises a complex interaction of environmental factors associated with genetic variation; however, the involved pathways are unclear. The identification of mutations in some genes associated with the PD has emerged as a possible cause for the disease pathogenesis. The hereditary mutation in six different genes, such as the synaptic protein a-synuclein [93] and E3 ubiquitin ligase, parkin [94] is associated with some PD forms.

Currently, dopamine replacement is the standard clinical treatment for PD patients, in order to ameliorate the motor symptoms. Therefore, the discovery of new therapies is crucial to improve both motor and non-motor symptoms, as the cognitive impairment and the autonomic nervous system dysfunction [91]. The transcription factor Nrf2 has emerged as a possible target to modulate the PD molecular hallmarks since it is able to regulate the proteasome and autophagy processes. Thus, Nrf2 modulation could be an alternative for the PD treatment.

Studies show a correlative decline in Nrf2 activity with age (the predominant risk factor for PD), suggesting an indirect link between Nrf2 and the disease [95,96]. Moreover, evidence has been published demonstrating that a deficient Nrf2-mediated antioxidant response is associated with oxidative stress, common in PD patients. In dopaminergic neurons from the substantia nigra pars compacta (SNpc), Nrf2 is usually located in the cytosol, whereas in PD patients, it is found in the nucleous [85]. Moreover, the up-regulated expression of NQO1 [97] and HO-1 [98,99,100,101] induced by Nrf2 suggests a brain protection through this mechanism [102]. A study regarding the expression of Nrf2, NQO1, and p62 in postmortem samples of PD patients showed an impaired neuroprotective capacity of this pathway [103].

However, a strong evidence regarding the association between Nrf2 and PD was demonstrated in a European case- control groups study. The results showed that a functional haplotype in the human NFE2L2 (Nuclear Factor, Erythroid 2 Like 2) gene promoter was associated with both decreased risk and delayed age at disease onset [104,105]. Several SNPs (single nucleotide polymorphism) have been identified as able to reduce the PD susceptibility in some conditions, as the regular exposure to pesticides [106].

The literature also reports some mutations in familiar condition of PD, such as leucine-rich repeat kinase 2 (LRRK2) gene mutations, emphasizing that it could be also considered useful as biomarkers. A strong positive correlation was found between Nrf2 and the Unified Parkinson’s Disease Rating Scale (UPDRS) in LRRK2-PD patients [107]. An in vitro study with induced pluripotent stem cells (iPSCs) from PARK2 (parkin gene) demonstrated increased oxidative stress and improved Nrf2 activity, which was correlated with changes in mitochondrial morphology and impaired mitochondrial homeostasis [108].

Nrf2 is also connected to PD by the protein deglycase DJ-1. Evidence shows that a mutation in DJ-1 induces an early familial form of PD [109]. Moreover, it has been demonstrated that DJ-1 has an important role in the NRF2-dependent oxidative stress response, up-regulating 20S proteasome and its regulator, NQO1 [110]. Moreover, DJ-1 is able to induce thioredoxin 1 expression through NRF2 pathway [111] and also stabilizes Nrf2 avoiding its ubiquitination and degradation [112]. Corroborating this effect, an experimental PD model using DJ-1/-mice did not show neuronal loss [113,114]; however, the neurons presented more susceptibility to toxic insults [113], demonstrating a similar pattern in DJ-1/- and Nrf2/-mice [115], explained by the loss of the antioxidant gene transcription. Dopaminergic neuron loss in Nrf2/-mice was associated with increased neuroinflammation, demonstrating an important role of Nrf2 to regulate neurodegenerative and neuroinflammatory processes [98].

α-synuclein was demonstrated to induce antioxidant enzyme genes in microglial cells via Nrf2. A study showing that misfolded α-synuclein directly activates microglia and increased antioxidant enzyme expression corroborate this finding [116]. Mice that overexpress Nrf2 and human mutant α-synuclein in neurons demonstrated an extended life span, increased motor neuron survival, and reduced oxidative stress compared to mutant α-synuclein (A53T) mice [117]. In vitro study with SK-N-SH cells showed α-synuclein aggregation and neurotoxicity by NRF2/HO-1 inhibition induced by ferrous iron [118], suggesting an important role of NRF2 in PD.

**Figure 2:**
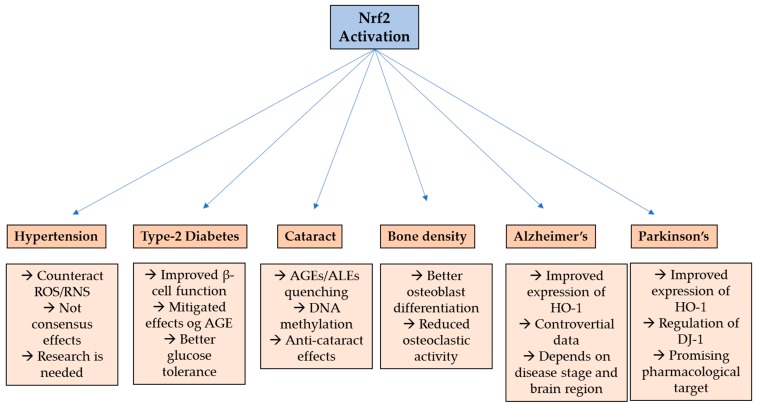
Nrf2 effect on age-related disorders. ROS—reactive oxygen species; RNS—reactive nitrogen species; AGE—advanced glycation end products; ALE—advanced lipoxidation end products; HO-1—heme oxygenase-1; DJ-1—protein deglycase.

In summary, Nrf2 has demonstrated interesting effects in age-related diseases (Figure 2) and a promise pharmacological target for PD patients, since many studies show a consistent role of this transcriptional factor to modulate for delaying the disease progression.

## 8. Conclusions

It is clear that Nrf2 exerts several functions in many conditions and diseases. Due the heterogeneity, it is impossible to concept that Nrf2 is a target to counteract aging. However, for some aging-related diseases it seems that the Nrf2 activation constitutes an interesting strategy. There is consistent evidence showing the beneficial effects of Nrf2 activation on pathophysiological processes of type-2 diabetes, and Alzheimer’s and Parkinson’s diseases. Together with positive evidence, strategies targeting Nrf2 are being investigated on these conditions and future effective therapies must be developed. Although studies with other age-related diseases do not demonstrate solid evidence, more research is still needed for better understanding Nrf2 activation and this pathway will still be an interesting focus in the coming years.
